# 9-Meth­oxy-5-phenyl­sulfonyl-5*H*-benzo[*b*]carbazole

**DOI:** 10.1107/S1600536808024756

**Published:** 2008-08-06

**Authors:** G. Chakkaravarthi, V. Dhayalan, A. K. Mohanakrishnan, V. Manivannan

**Affiliations:** a Department of Physics, CPCL Polytechnic College, Chennai 600 068, India; b Department of Organic Chemistry, University of Madras, Guindy Campus, Chennai 600 025, India; c Department of Physics, Presidency College, Chennai 600 005, India

## Abstract

In the title compound, C_23_H_17_NO_3_S, the mean plane of the benzo[*b*]carbazole ring system makes a dihedral angle of 77.17 (4)° with the phenyl ring. The S atom is in a distorted tetra­hedral configuration. There are three intra­molecular C—H⋯O inter­actions forming five- and six-membered rings with graph-set motifs *S*(5) and *S*(6), respectively.

## Related literature

For related literature, see: Allen *et al.* (1987 ▶); Chakkaravarthi *et al.* (2007 ▶, 2008 ▶); Diaz *et al.* (2002 ▶); Etter *et al.* (1990 ▶); Govindasamy *et al.* (1998 ▶); Hökelek *et al.* (1998 ▶); Hosomi *et al.* (2000 ▶); Itoigawa *et al.* (2000 ▶); Ramsewak *et al.* (1999 ▶); Rodriguez *et al.* (1995 ▶); Sankaranarayanan *et al.* (2000 ▶); Tachibana *et al.* (2001 ▶); Zhang *et al.* (2004 ▶).
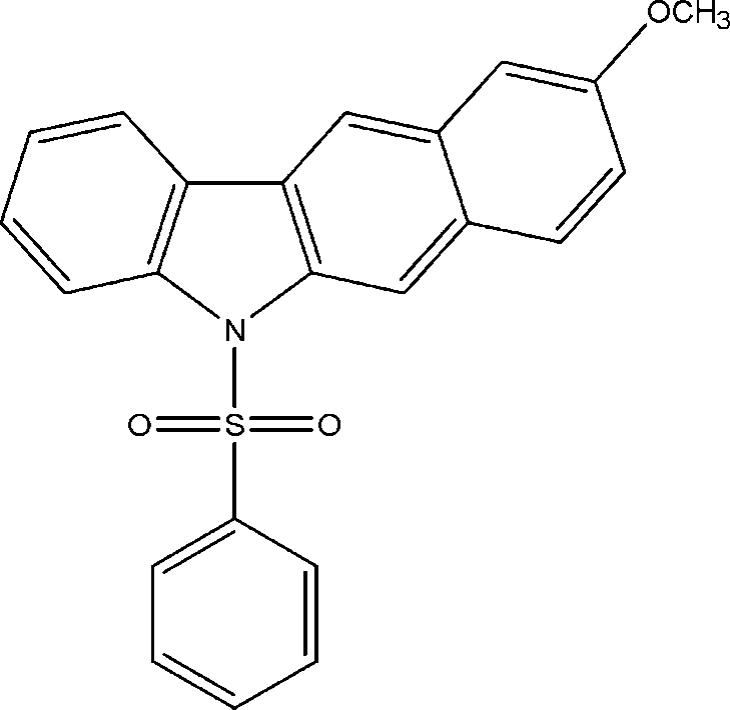


## Experimental

### 

#### Crystal data

C_23_H_17_NO_3_S
*M* *_r_* = 387.44
Triclinic, 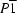

*a* = 8.3608 (3) Å
*b* = 9.3103 (3) Å
*c* = 12.1754 (4) Å
α = 76.061 (2)°
β = 88.680 (1)°
γ = 88.715 (2)°
*V* = 919.46 (5) Å^3^
*Z* = 2
Mo *K*α radiation
μ = 0.20 mm^−1^
*T* = 295 (2) K
0.30 × 0.20 × 0.16 mm

#### Data collection

Bruker Kappa APEX2 diffractometer
Absorption correction: multi-scan (*SADABS*; Sheldrick, 1996 ▶) *T*_min_ = 0.909, *T*_max_ = 0.969
23200 measured reflections
5739 independent reflections
4330 reflections with *I* > 2σ(*I*)
*R*_int_ = 0.022

#### Refinement

*R*[*F* ^2^ > 2σ(*F* ^2^)] = 0.042
*wR*(*F* ^2^) = 0.134
*S* = 1.05
5739 reflections
254 parameters
H-atom parameters constrained
Δρ_max_ = 0.28 e Å^−3^
Δρ_min_ = −0.35 e Å^−3^

### 

Data collection: *APEX2* (Bruker, 2004 ▶); cell refinement: *APEX2*; data reduction: *APEX2*; program(s) used to solve structure: *SHELXS97* (Sheldrick, 2008 ▶); program(s) used to refine structure: *SHELXL97* (Sheldrick, 2008 ▶); molecular graphics: *PLATON* (Spek, 2003 ▶); software used to prepare material for publication: *SHELXL97*.

## Supplementary Material

Crystal structure: contains datablocks I, global. DOI: 10.1107/S1600536808024756/bt2761sup1.cif

Structure factors: contains datablocks I. DOI: 10.1107/S1600536808024756/bt2761Isup2.hkl

Additional supplementary materials:  crystallographic information; 3D view; checkCIF report

## Figures and Tables

**Table 1:** Hydrogen-bond geometry (Å, °)

*D*—H⋯*A*	*D*—H	H⋯*A*	*D*⋯*A*	*D*—H⋯*A*
C8—H8⋯O1	0.93	2.36	2.951 (2)	121
C21—H21⋯O2	0.93	2.36	2.9460 (18)	121
C6—H6⋯O1	0.93	2.54	2.906 (2)	104

**Fig. 1.**
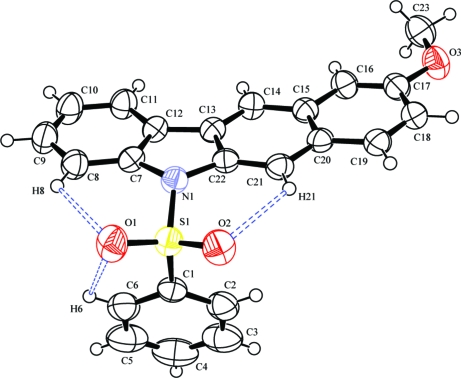
The molecular structure of (I), with atom labeling scheme. Displacement ellipsoids are drawn at 50% probability level. H atoms are presented as a small spheres of arbitrary radius. Intramolecular H-bonds are shown as dashed lines.

## supplementary crystallographic information

### Comment

Carbazole derivatives exhibit antitumor (Itoigawa *et al.*, 2000), antioxidative (Tachibana *et al.*, 2001), anti-inflammatory and antimutagenic (Ramsewak *et al.*, 1999) activities. These compounds are thermally and photochemically stable, which makes them useful materials for technological applications. For instance, the carbazole ring is easily funtionalized and covalently linked to other molecules (Diaz *et al.*, 2002). This enables its use as a convenient building block for the design and synthesis of molecular glasses, which are widely studied as components of electroactive and photoactive materials (Zhang *et al.*, 2004).

The geometric parameters in (I), (Fig. 1) agree with the reported similiar structures (Hökelek *et al.*, 1998; Hosomi *et al.*, 2000). The mean planes of the benzo[*b*]carbazole and phenyl ring form a dihedral angle of 77.17 (4)°. The N1—S1—C1 plane is almost orthogonal to carbazole ring [dihedral angle 89.54 (5)°] and phenyl ring [dihedral angle 86.02 (6)°]. The best plane of pyrrole ring N1/C7/C12/C13/C22 subtends a dihedral angle of 30.13 (8)° with sulfonyl group.

The average S—O, S—C, and S—N distances are comparable with those observed in similiar structures (Chakkaravarthi *et al.*, 2007; Sankaranarayanan *et al.*, 2000). The N—C bond lengths, namely N1—C7 and N1—C22 [1.4352 (17) & 1.4340 (16) Å] deviate slightly from the normal mean value reported in the literature (Allen *et al.*, 1987). This indicates that the substitution of the phenylsulfonyl group at atom N1 results in lengthening of the C—N bond lengths. This may be due to the electron-withdrawing character of the phenylsulfonyl group (Govindasamy *et al.*, 1998).

The S atom exhibits significant deviation from   a regular tetrahedron, with the largest deviations being seen for the O1—S1—O2 [120.09 (9)°] and O1—S1—N1 [106.78 (7)°] angles. The widening of the angles may be due to repulsive interactions between the two short S=O bonds, similar to what is observed in related structures (Chakkaravarthi *et al.*, 2008; Rodriguez *et al.*, 1995). The sum of the bond angles around N1 [351.97°] indicate the *sp*^2^ hybridized state of the atom N, in the molecule.

The benzene ring C15—C20 is almost coplanar with methoxy group [torsion angle C23—O3—C17—C16 5.4 (2)°]. The torsion angles O1—S1—N1—C7 and O2—S1—N1—C22 [-44.62 (12)° and 41.56 (12)°, respectively] describe the *syn* conformation of the phenylsulfonyl group with respect to benzocarbazole ring system. This conformation is influenced by the intramolecular C—H··· O hydrogen bonds, C8—H8··· O1 and C21—H21···O2, involving sulfonyl atoms O1 and O2 (Table 1). The intramolecular hydrogen bonds form a six-membered ring  with a graph-set motif of S(6) and   a five-membered ring  with a graph-set motif of S(5) (Etter *et al.*, 1990).

### Experimental

To a solution of diethyl 2-((2-(bromomethyl)-1-(phenylsulfonyl)-1*H*-indol-3-yl)methylene) malonate (0.57 mmol) in dry 1,2-DCE (10 ml), ZnBr_2_ (1.15 mmol) and anisole (1.15 mmol) were added. The reaction mixture was then refluxed for 1 h under N_2_ atmosphere. It was then poured over ice-water (30 ml) containing 1 ml of concentrated HCl, extracted with Chloroform (2 *X* 10 ml) and dried (Na_2_SO_4_). The solvent was removed under vacuo, then crude products was purified by flash column chromatography (silica gel, 230–420 mesh, n-hexane/ethyl acetate 98:2) afforded the title compound   suitable for X-ray analysis.

### Refinement

H atoms were positioned geometrically and refined using a riding model with C—H = 0.93 Å and *U*_iso_(H) = 1.2Ueq(C) for aromatic H atoms and C—H = 0.96 Å and *U*_iso_(H) = 1.5Ueq(C) for methyl H atoms.

### Crystal data

**Table:**

C_23_H_17_NO_3_S	*Z* = 2
*M**_r_* = 387.44	*F*_000_ = 404
Triclinic, *P*1	*D*_x_ = 1.399 Mg m^−^^3^
Hall symbol: -P 1	Mo *K*α radiation λ = 0.71073 Å
*a* = 8.3608 (3) Å	Cell parameters from 6534 reflections
*b* = 9.3103 (3) Å	θ = 2.4–30.7º
*c* = 12.1754 (4) Å	µ = 0.20 mm^−^^1^
α = 76.061 (2)º	*T* = 295 (2) K
β = 88.6800 (10)º	Block, colourless
γ = 88.715 (2)º	0.30 × 0.20 × 0.16 mm
*V* = 919.46 (5) Å^3^	

### Data collection

**Table:**

Bruker Kappa APEX2 diffractometer	5739 independent reflections
Radiation source: fine-focus sealed tube	4330 reflections with *I* > 2σ(*I*)
Monochromator: graphite	*R*_int_ = 0.022
*T* = 295(2) K	θ_max_ = 30.9º
ω and φ scans	θ_min_ = 2.3º
Absorption correction: multi-scan(SADABS; Sheldrick, 1996)	*h* = −12→12
*T*_min_ = 0.909, *T*_max_ = 0.969	*k* = −12→13
23200 measured reflections	*l* = −17→17

### Refinement

**Table:**

Refinement on *F*^2^	Secondary atom site location: difference Fourier map
Least-squares matrix: full	Hydrogen site location: inferred from neighbouring sites
*R*[*F*^2^ > 2σ(*F*^2^)] = 0.042	H-atom parameters constrained
*wR*(*F*^2^) = 0.134	*w* = 1/[σ^2^(*F*_o_^2^) + (0.0653*P*)^2^ + 0.1785*P*] where *P* = (*F*_o_^2^ + 2*F*_c_^2^)/3
*S* = 1.05	(Δ/σ)_max_ < 0.001
5739 reflections	Δρ_max_ = 0.29 e Å^−^^3^
254 parameters	Δρ_min_ = −0.35 e Å^−^^3^
Primary atom site location: structure-invariant direct methods	Extinction correction: none

### Fractional atomic coordinates and isotropic or equivalent isotropic displacement parameters (Å^2^)

**Table:**

	*x*	*y*	*z*	*U*_iso_*/*U*_eq_	
S1	0.46793 (4)	0.47800 (4)	0.77672 (3)	0.04613 (11)	
O1	0.54144 (15)	0.52427 (14)	0.66788 (10)	0.0629 (3)	
O2	0.55193 (14)	0.49026 (13)	0.87398 (10)	0.0595 (3)	
O3	0.19450 (16)	−0.22782 (14)	1.37354 (9)	0.0650 (3)	
N1	0.42588 (14)	0.30123 (12)	0.79458 (9)	0.0434 (2)	
C1	0.28152 (18)	0.56690 (14)	0.77467 (12)	0.0473 (3)	
C2	0.2064 (2)	0.57404 (18)	0.87596 (15)	0.0616 (4)	
H2	0.2572	0.5391	0.9447	0.074*	
C3	0.0533 (3)	0.6349 (2)	0.8715 (2)	0.0820 (6)	
H3	−0.0003	0.6411	0.9380	0.098*	
C4	−0.0204 (3)	0.6865 (2)	0.7683 (3)	0.0895 (7)	
H4	−0.1243	0.7246	0.7663	0.107*	
C5	0.0581 (3)	0.6822 (2)	0.6692 (2)	0.0845 (7)	
H5	0.0085	0.7199	0.6004	0.101*	
C6	0.2093 (2)	0.62243 (18)	0.67116 (15)	0.0635 (4)	
H6	0.2631	0.6191	0.6041	0.076*	
C7	0.35493 (16)	0.24535 (15)	0.70734 (11)	0.0419 (3)	
C8	0.37507 (19)	0.29073 (17)	0.59112 (12)	0.0512 (3)	
H8	0.4363	0.3725	0.5579	0.061*	
C9	0.3005 (2)	0.20949 (19)	0.52609 (13)	0.0578 (4)	
H9	0.3115	0.2377	0.4477	0.069*	
C10	0.2101 (2)	0.0875 (2)	0.57509 (13)	0.0602 (4)	
H10	0.1620	0.0347	0.5294	0.072*	
C11	0.1905 (2)	0.04329 (18)	0.69090 (13)	0.0540 (4)	
H11	0.1295	−0.0388	0.7237	0.065*	
C12	0.26309 (16)	0.12316 (15)	0.75771 (11)	0.0427 (3)	
C13	0.27196 (15)	0.10092 (14)	0.87926 (11)	0.0409 (3)	
C14	0.20944 (17)	−0.00631 (16)	0.96676 (12)	0.0457 (3)	
H14	0.1427	−0.0777	0.9521	0.055*	
C15	0.24777 (16)	−0.00676 (15)	1.07919 (11)	0.0428 (3)	
C16	0.19098 (19)	−0.11947 (17)	1.17162 (12)	0.0504 (3)	
H16	0.1228	−0.1911	1.1589	0.060*	
C17	0.23668 (18)	−0.12252 (17)	1.27913 (12)	0.0498 (3)	
C18	0.33749 (19)	−0.01305 (17)	1.29944 (12)	0.0516 (3)	
H18	0.3674	−0.0160	1.3731	0.062*	
C19	0.39146 (19)	0.09670 (16)	1.21281 (12)	0.0486 (3)	
H19	0.4572	0.1686	1.2280	0.058*	
C20	0.34939 (16)	0.10378 (14)	1.09928 (11)	0.0419 (3)	
C21	0.41158 (17)	0.21431 (15)	1.00847 (12)	0.0444 (3)	
H21	0.4774	0.2874	1.0216	0.053*	
C22	0.37207 (15)	0.21053 (14)	0.90096 (11)	0.0398 (3)	
C23	0.1076 (2)	−0.3496 (2)	1.35768 (15)	0.0671 (5)	
H23A	0.0031	−0.3164	1.3303	0.101*	
H23B	0.0974	−0.4218	1.4284	0.101*	
H23C	0.1632	−0.3932	1.3036	0.101*	

### Atomic displacement parameters (Å^2^)

**Table:**

	*U*^11^	*U*^22^	*U*^33^	*U*^12^	*U*^13^	*U*^23^
S1	0.0454 (2)	0.04688 (19)	0.04625 (18)	−0.01263 (14)	0.00349 (13)	−0.01082 (13)
O1	0.0640 (7)	0.0674 (7)	0.0561 (6)	−0.0223 (6)	0.0174 (5)	−0.0124 (5)
O2	0.0592 (7)	0.0604 (6)	0.0604 (6)	−0.0184 (5)	−0.0094 (5)	−0.0151 (5)
O3	0.0758 (8)	0.0647 (7)	0.0478 (6)	−0.0110 (6)	−0.0011 (5)	0.0005 (5)
N1	0.0449 (6)	0.0423 (5)	0.0435 (5)	−0.0045 (5)	0.0008 (4)	−0.0115 (4)
C1	0.0536 (8)	0.0348 (6)	0.0532 (7)	−0.0069 (5)	0.0004 (6)	−0.0094 (5)
C2	0.0682 (10)	0.0485 (8)	0.0638 (9)	−0.0045 (7)	0.0141 (8)	−0.0062 (7)
C3	0.0766 (13)	0.0555 (10)	0.1082 (17)	0.0014 (9)	0.0332 (12)	−0.0121 (10)
C4	0.0642 (12)	0.0570 (11)	0.145 (2)	0.0128 (9)	−0.0064 (14)	−0.0210 (13)
C5	0.0874 (15)	0.0581 (11)	0.1115 (18)	0.0186 (10)	−0.0349 (14)	−0.0258 (11)
C6	0.0789 (12)	0.0500 (8)	0.0634 (9)	0.0034 (8)	−0.0149 (8)	−0.0160 (7)
C7	0.0396 (6)	0.0440 (6)	0.0445 (6)	0.0008 (5)	0.0015 (5)	−0.0154 (5)
C8	0.0554 (8)	0.0526 (8)	0.0460 (7)	−0.0061 (6)	0.0064 (6)	−0.0129 (6)
C9	0.0680 (10)	0.0649 (9)	0.0428 (7)	−0.0058 (8)	0.0028 (6)	−0.0177 (6)
C10	0.0701 (11)	0.0673 (10)	0.0492 (8)	−0.0115 (8)	−0.0030 (7)	−0.0243 (7)
C11	0.0595 (9)	0.0555 (8)	0.0503 (7)	−0.0133 (7)	0.0001 (6)	−0.0182 (6)
C12	0.0406 (7)	0.0464 (7)	0.0429 (6)	−0.0011 (5)	0.0010 (5)	−0.0146 (5)
C13	0.0369 (6)	0.0435 (6)	0.0434 (6)	0.0003 (5)	−0.0011 (5)	−0.0129 (5)
C14	0.0434 (7)	0.0473 (7)	0.0474 (7)	−0.0078 (5)	−0.0011 (5)	−0.0127 (5)
C15	0.0395 (6)	0.0437 (6)	0.0445 (6)	0.0013 (5)	−0.0003 (5)	−0.0098 (5)
C16	0.0492 (8)	0.0508 (7)	0.0490 (7)	−0.0055 (6)	0.0005 (6)	−0.0076 (6)
C17	0.0495 (8)	0.0499 (7)	0.0464 (7)	0.0041 (6)	0.0003 (6)	−0.0051 (6)
C18	0.0587 (9)	0.0516 (8)	0.0447 (7)	0.0086 (6)	−0.0078 (6)	−0.0121 (6)
C19	0.0534 (8)	0.0458 (7)	0.0486 (7)	0.0031 (6)	−0.0076 (6)	−0.0149 (6)
C20	0.0410 (7)	0.0411 (6)	0.0446 (6)	0.0053 (5)	−0.0027 (5)	−0.0126 (5)
C21	0.0455 (7)	0.0404 (6)	0.0490 (7)	−0.0019 (5)	−0.0039 (5)	−0.0133 (5)
C22	0.0373 (6)	0.0385 (6)	0.0438 (6)	0.0012 (5)	0.0000 (5)	−0.0107 (5)
C23	0.0704 (11)	0.0616 (10)	0.0617 (10)	−0.0096 (8)	0.0024 (8)	0.0004 (8)

### Geometric parameters (Å, °)

**Table:**

S1—O2	1.4197 (11)	C10—C11	1.377 (2)
S1—O1	1.4208 (11)	C10—H10	0.9300
S1—N1	1.6521 (12)	C11—C12	1.385 (2)
S1—C1	1.7461 (16)	C11—H11	0.9300
O3—C17	1.3641 (17)	C12—C13	1.4475 (18)
O3—C23	1.414 (2)	C13—C14	1.3748 (18)
N1—C22	1.4340 (16)	C13—C22	1.4107 (18)
N1—C7	1.4352 (17)	C14—C15	1.4120 (19)
C1—C2	1.386 (2)	C14—H14	0.9300
C1—C6	1.388 (2)	C15—C20	1.418 (2)
C2—C3	1.385 (3)	C15—C16	1.4219 (19)
C2—H2	0.9300	C16—C17	1.366 (2)
C3—C4	1.386 (3)	C16—H16	0.9300
C3—H3	0.9300	C17—C18	1.407 (2)
C4—C5	1.369 (3)	C18—C19	1.356 (2)
C4—H4	0.9300	C18—H18	0.9300
C5—C6	1.368 (3)	C19—C20	1.4200 (19)
C5—H5	0.9300	C19—H19	0.9300
C6—H6	0.9300	C20—C21	1.4146 (19)
C7—C8	1.3833 (19)	C21—C22	1.3662 (18)
C7—C12	1.3933 (19)	C21—H21	0.9300
C8—C9	1.386 (2)	C23—H23A	0.9600
C8—H8	0.9300	C23—H23B	0.9600
C9—C10	1.381 (2)	C23—H23C	0.9600
C9—H9	0.9300		
			
O2—S1—O1	119.66 (7)	C10—C11—H11	120.6
O2—S1—N1	106.69 (6)	C12—C11—H11	120.6
O1—S1—N1	106.78 (7)	C11—C12—C7	119.86 (13)
O2—S1—C1	109.52 (7)	C11—C12—C13	131.94 (13)
O1—S1—C1	108.60 (8)	C7—C12—C13	108.10 (12)
N1—S1—C1	104.52 (6)	C14—C13—C22	120.69 (12)
C17—O3—C23	117.30 (13)	C14—C13—C12	131.56 (13)
C22—N1—C7	107.12 (10)	C22—C13—C12	107.65 (11)
C22—N1—S1	122.77 (9)	C13—C14—C15	119.27 (13)
C7—N1—S1	122.08 (9)	C13—C14—H14	120.4
C2—C1—C6	121.82 (16)	C15—C14—H14	120.4
C2—C1—S1	119.46 (12)	C14—C15—C20	119.23 (12)
C6—C1—S1	118.64 (13)	C14—C15—C16	120.91 (13)
C3—C2—C1	117.85 (18)	C20—C15—C16	119.81 (13)
C3—C2—H2	121.1	C17—C16—C15	119.97 (14)
C1—C2—H2	121.1	C17—C16—H16	120.0
C2—C3—C4	120.2 (2)	C15—C16—H16	120.0
C2—C3—H3	119.9	O3—C17—C16	125.14 (15)
C4—C3—H3	119.9	O3—C17—C18	114.46 (13)
C5—C4—C3	120.8 (2)	C16—C17—C18	120.40 (14)
C5—C4—H4	119.6	C19—C18—C17	120.70 (14)
C3—C4—H4	119.6	C19—C18—H18	119.6
C6—C5—C4	120.1 (2)	C17—C18—H18	119.6
C6—C5—H5	119.9	C18—C19—C20	121.16 (14)
C4—C5—H5	119.9	C18—C19—H19	119.4
C5—C6—C1	119.11 (19)	C20—C19—H19	119.4
C5—C6—H6	120.4	C21—C20—C15	120.89 (12)
C1—C6—H6	120.4	C21—C20—C19	121.12 (13)
C8—C7—C12	121.66 (13)	C15—C20—C19	117.95 (13)
C8—C7—N1	129.40 (13)	C22—C21—C20	118.05 (13)
C12—C7—N1	108.81 (11)	C22—C21—H21	121.0
C7—C8—C9	117.38 (14)	C20—C21—H21	121.0
C7—C8—H8	121.3	C21—C22—C13	121.87 (12)
C9—C8—H8	121.3	C21—C22—N1	129.70 (12)
C10—C9—C8	121.48 (14)	C13—C22—N1	108.31 (11)
C10—C9—H9	119.3	O3—C23—H23A	109.5
C8—C9—H9	119.3	O3—C23—H23B	109.5
C11—C10—C9	120.74 (15)	H23A—C23—H23B	109.5
C11—C10—H10	119.6	O3—C23—H23C	109.5
C9—C10—H10	119.6	H23A—C23—H23C	109.5
C10—C11—C12	118.87 (14)	H23B—C23—H23C	109.5
			
O2—S1—N1—C22	41.56 (12)	C11—C12—C13—C14	−0.7 (3)
O1—S1—N1—C22	170.62 (11)	C7—C12—C13—C14	−176.98 (14)
C1—S1—N1—C22	−74.41 (12)	C11—C12—C13—C22	175.59 (15)
O2—S1—N1—C7	−173.68 (10)	C7—C12—C13—C22	−0.70 (15)
O1—S1—N1—C7	−44.62 (12)	C22—C13—C14—C15	−0.6 (2)
C1—S1—N1—C7	70.35 (11)	C12—C13—C14—C15	175.24 (13)
O2—S1—C1—C2	−29.51 (14)	C13—C14—C15—C20	0.2 (2)
O1—S1—C1—C2	−161.84 (12)	C13—C14—C15—C16	−177.31 (13)
N1—S1—C1—C2	84.48 (13)	C14—C15—C16—C17	176.37 (13)
O2—S1—C1—C6	153.51 (12)	C20—C15—C16—C17	−1.2 (2)
O1—S1—C1—C6	21.18 (14)	C23—O3—C17—C16	5.4 (2)
N1—S1—C1—C6	−92.51 (13)	C23—O3—C17—C18	−173.41 (14)
C6—C1—C2—C3	1.9 (2)	C15—C16—C17—O3	−177.59 (14)
S1—C1—C2—C3	−174.97 (13)	C15—C16—C17—C18	1.2 (2)
C1—C2—C3—C4	−0.1 (3)	O3—C17—C18—C19	178.60 (14)
C2—C3—C4—C5	−1.8 (3)	C16—C17—C18—C19	−0.3 (2)
C3—C4—C5—C6	1.9 (3)	C17—C18—C19—C20	−0.6 (2)
C4—C5—C6—C1	−0.1 (3)	C14—C15—C20—C21	0.4 (2)
C2—C1—C6—C5	−1.9 (3)	C16—C15—C20—C21	177.97 (12)
S1—C1—C6—C5	175.06 (14)	C14—C15—C20—C19	−177.30 (12)
C22—N1—C7—C8	−176.94 (14)	C16—C15—C20—C19	0.3 (2)
S1—N1—C7—C8	33.57 (19)	C18—C19—C20—C21	−177.08 (13)
C22—N1—C7—C12	−1.03 (14)	C18—C19—C20—C15	0.6 (2)
S1—N1—C7—C12	−150.52 (10)	C15—C20—C21—C22	−0.6 (2)
C12—C7—C8—C9	−0.2 (2)	C19—C20—C21—C22	177.01 (12)
N1—C7—C8—C9	175.27 (14)	C20—C21—C22—C13	0.2 (2)
C7—C8—C9—C10	−0.3 (3)	C20—C21—C22—N1	−175.36 (12)
C8—C9—C10—C11	0.4 (3)	C14—C13—C22—C21	0.4 (2)
C9—C10—C11—C12	−0.1 (3)	C12—C13—C22—C21	−176.35 (12)
C10—C11—C12—C7	−0.4 (2)	C14—C13—C22—N1	176.82 (12)
C10—C11—C12—C13	−176.35 (15)	C12—C13—C22—N1	0.06 (14)
C8—C7—C12—C11	0.5 (2)	C7—N1—C22—C21	176.62 (13)
N1—C7—C12—C11	−175.75 (13)	S1—N1—C22—C21	−34.15 (19)
C8—C7—C12—C13	177.36 (13)	C7—N1—C22—C13	0.58 (14)
N1—C7—C12—C13	1.07 (15)	S1—N1—C22—C13	149.81 (10)

### Hydrogen-bond geometry (Å, °)

**Table:**

*D*—H···*A*	*D*—H	H···*A*	*D*···*A*	*D*—H···*A*
C8—H8···O1	0.93	2.36	2.951 (2)	121
C21—H21···O2	0.93	2.36	2.9460 (18)	121
C6—H6···O1	0.93	2.54	2.906 (2)	104
